# Investigation of the Gelation Process of a Polymer Composition Based on an Acrylic Polymer

**DOI:** 10.3390/gels12030204

**Published:** 2026-02-28

**Authors:** Inzir Raupov, Tatiana Nosenko, Victoria Grigoreva, Vasiliy Zazulya, Gennadiy Sukhoroslov, Vyacheslav Shkodkin

**Affiliations:** 1 Department of Oil and Gas Fields Development and Operation, Empress Catherine II Saint Petersburg Mining University, 199106 Saint Petersburg, Russia; raupov_ir@pers.spmi.ru (I.R.);; 2 Chemical Engineering Centre, ITMO University, 197101 Saint Petersburg, Russia

**Keywords:** enhanced oil recovery, conformance control, crosslinked polymer compositions, polyacrylamide, gelation, rheology

## Abstract

The aim of this work is to describe the gelation process of a crosslinked polymer composition depending on its flow rate in free space and in pore space. The object of the study is a polymer solution based on partially hydrolyzed polyacrylonitrile and chromium acetate. A team of researchers has proposed a new approach to describing the kinetic viscosity curve of a crosslinked polymer system in a free volume. This approach takes into account oscillatory variations in the structural and mechanical characteristics relative to a smoothly increasing gelation curve. The nonlinear effects are linked to the processes of structural formation and, simultaneously, destruction due to mechanical, thermobaric, and chemical destruction under varying flow conditions. The proposed solution is based on the Verhulst differential equation and tested on five values of shear rates with the addition of correction factors. The article explains the processes of gel formation and the destruction of polymer compounds, and compares the equation of limited growth with the Kenneth Sorbie method, which is employed in the PC-GEL simulator. The limitations of the modern gel invasion model used in the UTCHEM and BPOPE simulator within the porous media (within a narrow gap) are revealed.

## 1. Introduction

The depletion of explored hydrocarbon reserves and the trend towards declining oil production rates necessitate the search for approaches and effective solutions to stabilize the falling production rates. Taking into account the specific features of financial investments in the mineral resources sector, namely the long payback period and large investment volumes required for prospecting, drilling, and development of new fields, the implementation of geological and technical measures to enhance oil recovery in developed fields presents an attractive solution [1]. A pressing issue in the development of brown oil fields with high geological heterogeneity is the formation of washed-out zones or preferential flow channels, through which the injected water flows into the production well, bypassing oil-saturated areas not covered by flooding [2,3,4].

One of the ways to solve this problem is the application of conformance control technology using a system of crosslinked polymers based on partially hydrolyzed polyacrylonitrile (PHPAN) and chromium acetate (CA) [5,6,7]. These crosslinked polymer systems are characterized by processes of structure formation, accompanied by gelation, as well as by destruction caused by mechanical, thermobaric, and chemical degradation under conditions of changing flow rates, temperature, pressure, adsorption, etc. [8,9,10]. Subsequently, these mechanisms lead to changes in the rheological properties of the composition, which in turn affects the efficiency of conformance control [11,12,13]. The rheological properties of solutions are also influenced by a large number of variable factors, including: the temperature, mineralization, component composition of the crosslinked polymer system and the concentration of each component, the viscoelastic properties of the crosslinked polymer system manifested during changes in flow rate within the porous media, polyelectrolyte effects, and thixotropic effects. These factors collectively determine the complex rheological behavior of the solutions, affecting their performance in various conditions [14,15,16]. The consideration of the factors outlined above in the flow processes of gel-forming compositions constitutes a complex and resource-intensive scientific challenge [17,18,19]. To determine the most optimal properties of a crosslinked polymer system and the technology for its injection into the reservoir, a comprehensive series of physicochemical studies of the compositions in free volume and filtration experiments on cores are conducted. Based on the results obtained hydrodynamic models are then developed [20,21,22].

The flow of gel-forming compositions has been the subject of studies by numerous researchers, addressing issues related to the description of the physicochemical properties of crosslinked polymer gels through the use of analytical models, the application of various polymer composition injection technologies, and gel modifications, taking into account the geological and technological conditions of field development, the positioning of the gel within the porous media, etc. [23,24,25]. However, many aspects of modeling the physicochemical properties of the compositions in question, such as the influence of component concentration, pore diameter, adsorption processes, and filtration rate on the kinetics of gelation reactions, as well as the analytical calculation of the porous media inaccessible to polymer molecules for filtration, still require further study [26,27,28].

The objective of the study is to describe the gelation process of a crosslinked polymer composition as a function of its flow rate in both free and pore spaces. The research focuses on the gelation process occurring in free space and within the porous media, with the object of the study being a polymer solution based on PHPAN and CA. The primary aim of the work is to identify and explain the dependencies between viscosity, gelation time, and shear rate.

## 2. Results and Discussion

### 2.1. Determination of Gel Concentration and Gelation Constant by Optical Method

**Figure 1:**
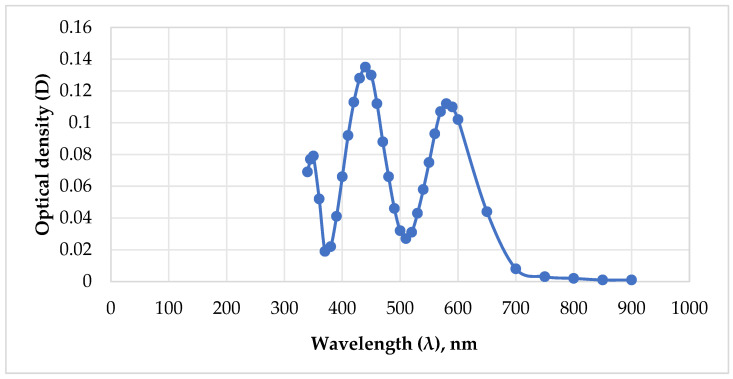
The dependence of the optical density of a CA solution on the wavelength.

**Figure 2:**
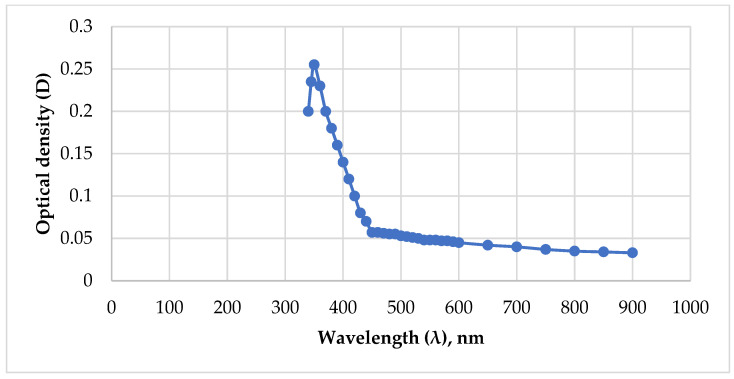
The dependence of the optical density of a PHPAN solution on the wavelength.

The authors proposed a method for measuring the gel concentration and determining the gelation constant based on the change in the optical density of the solution over time. The prepared aqueous solution of PHPAN and CA with concentrations of 0.7% and 0.07% was placed in a 10 mm wide cuvette, after which the optical density values of the gel relative to the comparison solution (comparison solution—CA with a concentration of 0.07%) were measured every 5 min using a Unico 2100 spectrophotometer (United Products & Instruments, Inc., Dayton, NJ, USA) at a wavelength of 350 nm. The wavelength was determined by identifying the maximum absorption values during optical studies of single-component solutions of PHPAN and CA with concentrations of 0.7% and 0.07% (Figure 1 and Figure 2). Analyzing the data of these dependencies, the highest light absorption is observed at 350 nm for PHPAN and CA at wavelengths of 350 nm, 420 nm, and 580 nm. Thus, the best light absorption for both gel components is observed only at a wavelength of 350 nm.

**Figure 3:**
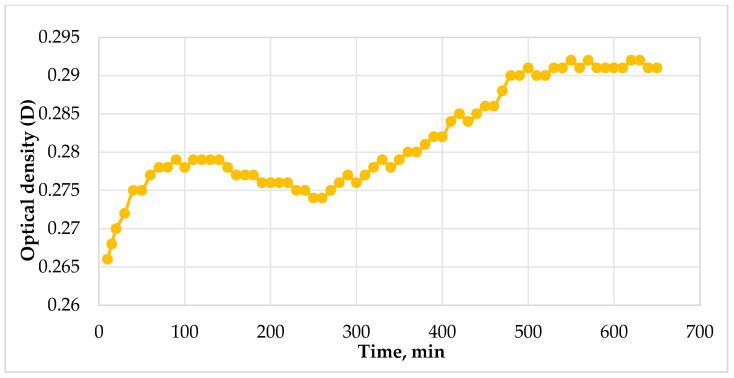
The dependence of the gel’s optical density on time.

The obtained dependence of the gel’s optical density on time is shown in Figure 3.

In accordance with the Beer–Lambert law (1), the absorption coefficients of the solutions of PHPAN (2) and CA (3) at the specified concentrations were calculated based on the results of the study of the dependence of optical density on wavelength:

(1) D=ε·C·l,

where D—optical density; ε—the absorption coefficient, 1/cm; C—the concentration of a substance, u.f.; l—the thickness of the substance layer, cm.

(2) $$\varepsilon_{PHPAN}=\frac{D_{PHPAN}}{C_{PHPAN}\cdot l}=\frac{0.255}{0.007\times 1}=36.43,$$

(3) $$\varepsilon_{CA}=\frac{D_{CA}}{C_{CA}\cdot l}=\frac{0.079}{0.0007\times 1}=112.86$$

From Figure 3, it can be concluded that the optical density reaches a predetermined value of 0.293, which corresponds to the completion of the gelation process.

The gelation mechanism of the studied polymer composition requires a more detailed analysis. The crosslinking process in the studied system is described within the framework of chromium(III) coordination chemistry. In aqueous acetate media, chromium(III) acetate does not behave as a simple ionic species but rather forms hydrolyzed and ligand-coordinated complexes capable of undergoing ligand substitution reactions. The coordination behavior of Cr^3+^ ions is well established in classical inorganic chemistry, where Cr(III) is recognized as a kinetically inert but thermodynamically stable metal center that preferentially interacts with oxygen-donor ligands [29]. Under aqueous conditions, acetate ligands may be partially displaced by stronger donor groups present in the polymer matrix, providing the chemical basis for network formation.

PHPAN contains several functional groups capable of participating in metal coordination. Depending on hydrolysis conditions, the polymer backbone may include carboxylate (–COO^−^), amide (–CONH_2_), and related oxygen-containing moieties, which serve as potential coordination sites. Chromium(III), as a hard Lewis acid with high charge density, predominantly coordinates through oxygen-containing ligands, making carboxylate groups the most probable binding centers. Such interactions may occur via monodentate, bidentate, or bridging coordination modes, ultimately leading to the formation of interchain coordination bridges. Similar mechanisms have been extensively described for chromium-crosslinked polyacrylamide and related polymer systems used in enhanced oil recovery and conformance control technologies. In particular, the comprehensive review [30] discusses multivalent-metal-induced crosslinking in aqueous polymer solutions and emphasizes the dominant role of coordination interactions in determining gel structure and rheological behavior.

From a mechanistic perspective, gelation can be interpreted as a sequence of ligand exchange and coordination processes. Initially, chromium(III) acetate complexes present in solution undergo partial ligand substitution, whereby acetate groups are replaced by polymer-bound donor ligands. This results in the progressive formation of Cr–O (polymer) coordination bonds and the emergence of interchain bridges. The relatively slow kinetics of ligand substitution characteristic of Cr(III) complexes provides a natural explanation for the experimentally observed gelation timescales. This interpretation is consistent with established studies of chromium-based polymer gels, including the works [31,32], which demonstrate that gelation kinetics in Cr(III)-crosslinked systems are controlled by coordination chemistry and ligand exchange processes.

Polymer gelation represents a network-forming transformation involving stochastic crosslinking events, evolving topology, and reaction–diffusion coupling. Classical chemical kinetics concepts, such as strict reaction order, are therefore of limited applicability. Theoretical treatments of polymer network formation, including those developed by Flory [33] and Rubinstein and Colby [34], highlight that macroscopic gelation behavior arises from collective structural evolution rather than discrete elementary reactions. In this context, the chromium-induced gelation observed in the present study is best understood as a coordination-driven assembly process.

In order to derive the dependence of gel concentration on time, several assumptions were made:

1. The entire PHPAN is crosslinked as a result of the gelation reaction;
2. At the end of the reaction, only the crosslinked polymer system and unreacted chromium acetate remain;
3. The change in the concentration of chromium acetate can be neglected due to its low initial concentration.

Thus, based on these assumptions, the absorption coefficient of the gel can be determined (4), and then the concentration values can be calculated from the optical density values (5):

(4) $$\varepsilon_{g}=\frac{D_{pred}}{C_{PHPAN}\cdot l}=\frac{0.293}{0.007\times 1}=41.86,$$

(5) $$C_{g}=\frac{D-D_{PHPAN}}{\varepsilon_{g}\cdot l}$$

where $D_{pred}$—predetermined value of the optical density; D—current value of optical density; $D_{PHPAN}$—optical density of the PHPAN; $\varepsilon_{g}$—absorption coefficient of the gel, 1/cm.

**Figure 4:**
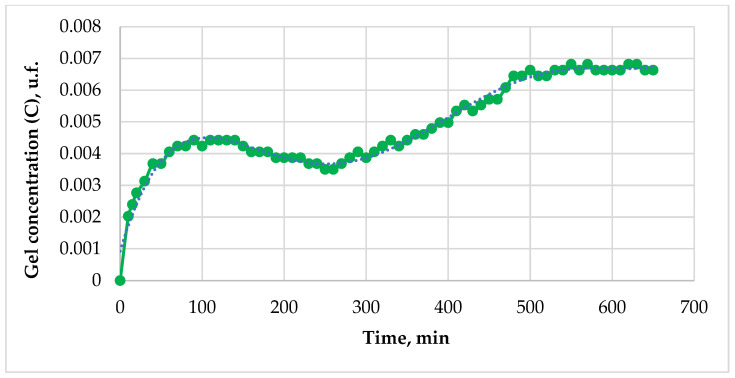
The dependence of gel concentration on time.

Based on the calculated gel concentration values, the dependence of gel concentration on time can be plotted (Figure 4).

To determine the gelation constant, it is necessary to linearize the graph of concentration as a function of time. The original graph correlates with a fifth-degree polynomial, which means that the gelation reaction follows a fifth-order kinetic equation. This approximation is exclusively phenomenological in nature and does not imply an elementary fifth-order reaction mechanism.

In this context, $K_{g}$ should be interpreted as an effective macroscopic kinetic parameter characterizing the rate of viscosity and structure evolution, rather than a strict reaction rate constant associated with a defined reaction order. This approach is consistent with the generally accepted principles of polymer system kinetics. The gelation process is considered a multistage physicochemical transformation involving coordination interactions, structural rearrangement of the macromolecular network, and diffusion effects. The kinetics of polymer crosslinking reactions are governed by the accessibility of functional groups, the growth of the local microstructure, and coupled reaction–diffusion processes. In the literature on polymer gelation and crosslinking [35], effective kinetic parameters are commonly used without assigning a formal reaction order.

We will solve the differential equation of the reaction rate of the fifth order using the law of mass action:

(6) $$-\frac{\partial C}{\partial t}=K_{g}\cdot C_{g}^{5},$$

Integrate both sides of the equation:

(7) $$-\int_{C_{0}}^{C}\frac{1}{C_{g}^{5}}dC=\int_{0}^{t}K_{g}dt,$$

Obtain the integral equation of the 4th order:

(8) $$\frac{1}{C_{0}^{4}}-\frac{1}{C^{4}}=4K_{g}t.$$

**Figure 5:**
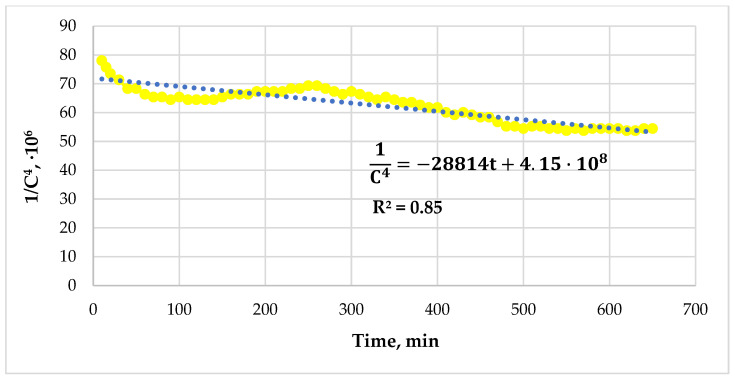
The linearized graph of the dependence of concentration on time.

Plot the graph of the dependence of $\frac{1}{C^{4}}$ on time (Figure 5). Then, the tangent of the angle between the line, whose equation is the trendline, and the time axis will be equal to −$4K_{g}$.

The gelation constant will be equal to

(9) $$K_{g}=\frac{\tan\alpha }{-4}=\frac{-28,814}{-4}=7203.5\;s^{-1}.$$

Importantly, the introduction of the gelation constant $K_{g}$ in this work serves a practical modeling purpose rather than a mechanistic one. The primary objective was to obtain an effective gelation constant enabling comparison with reservoir-scale gelation models, specifically the Kenneth Sorbie framework implemented in the PC-GEL simulator, which relies on the Arrhenius-type temperature dependence of gelation kinetics. Therefore, the derived constant $K_{g}$ is best understood as an effective kinetic descriptor suitable for comparative modeling and engineering analysis, not as proof of fifth-order reaction kinetics.

### 2.2. Application of the Verhulst Equation for Describing the Gelation Process in Free Volume

The PC-GEL and UTCHEM simulators are used to determine the rheological characteristics, such as the dependence of viscosity and gel concentration on time. These simulators use the following dependencies [13,36,37]:

(10) $$\mu =\mu_{0}+\alpha_{1}C_{g}+\alpha_{2}C_{g}^{2}+\alpha_{3}C_{g}^{3},$$

where μ—viscosity of the hydrogel, Pa·s; $\mu_{0}$—viscosity of the hydrogel at the moment t=0, Pa·s; $C_{g}$—gel concentration; $\alpha_{1},\;\alpha_{2},\;\alpha_{3}$—system constants.

(11) $$C_{g}=K_{g}C_{p}C_{Cr}t,$$

where $K_{g}$—the gelation rate constant, 1/s; $C_{p},\;C_{Cr}$—the concentrations of polymer and chromium acetate at the beginning of the experiment.

By substituting Equation (11) into Equation (10), we get

(12) $$\mu =\mu_{0}+\alpha_{1}K_{g}C_{p}C_{Cr}t+\alpha_{2}{\left(K_{g}C_{p}C_{Cr}\right)}^{2}t^{2}+\alpha_{3}{\left(K_{g}C_{p}C_{Cr}\right)}^{3}t^{3}.$$

**Figure 6:**
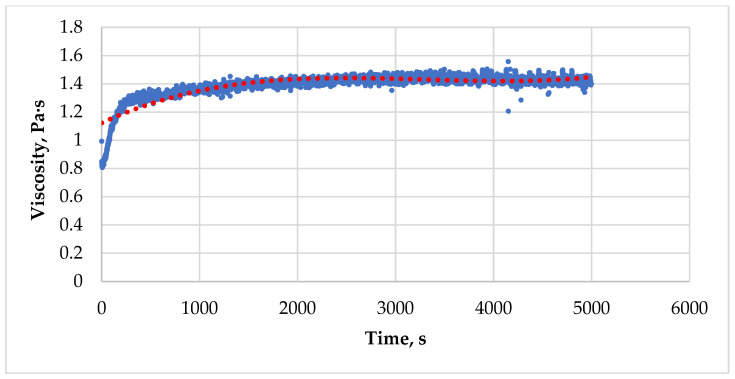
The dependence of viscosity on time of a PHPAN solution with CA according to the simulator PC-GEL at shear rate 3.82 s^−1^.

At the beginning of the experiment, the concentrations of polymer and chromium acetate, the initial viscosity value and the gelation rate constant are constant values. Therefore, in the PC-GEL model, the viscosity of the gel depends only on time, and the dependence has the form of a polynomial of the 3rd degree:

$\mu =1.1+0.3\cdot 10^{-3}t-10^{-7}t^{2}+10^{-11}t^{3}$ (Figure 6).

When approximating the experimental data using a third-degree polynomial equation, the correlation coefficient is 79%, which indicates a relatively weak correlation. A third-degree polynomial can describe the behavior of the gel in a limited range of the graph, but it does not fully reflect the gelation process throughout the entire study range. Therefore, we proposed an alternative model to describe the physical process of gelation.

**Figure 7:**
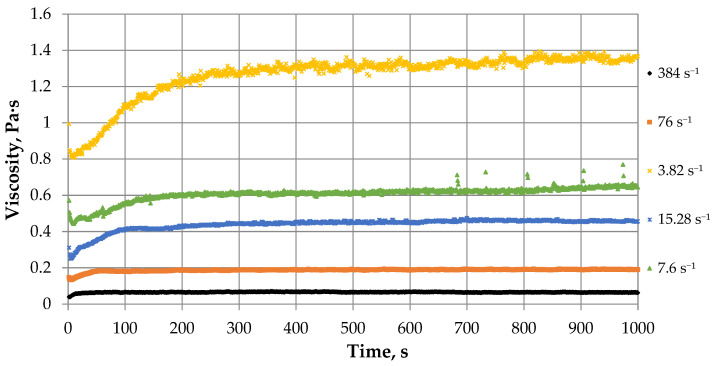
The dependence of viscosity on time of a PHPAN solution with CA at different shear rates.

During the analysis of the data on gelation time and the viscosity parameter over time (Figure 7), we obtained models of limited growth that can be approximated using the Verhulst equation:

(13) $$\frac{d\mu }{dt}=\mu r\cdot \left(1-\frac{\mu }{K}\right),$$

where K—steady viscosity value of the crosslinked gel, Pa·s; r—coefficient indicating the rate of viscosity growth, 1/s; t—time, s.

Let us transform Equation (13) into the form

(14) $$\left(\frac{1}{\mu }+\frac{1}{K-\mu }\right)d\mu =rdt.$$

Now, we integrate both sides of this equation:

(15) $$\int_{\mu_{0}}^{\mu }\left(\frac{1}{\mu }+\frac{1}{K-\mu }\right)d\mu =\int_{0}^{t}rdt.$$

Then we have

(16) $$\ln\left(\frac{\mu }{K-\mu }\right)-ln\left(\frac{\mu_{0}}{K-\mu_{0}}\right)=rt.$$

After some transformations, we will obtain a dependency:

(17) $$\mu \left(t\right)=\frac{K\mu_{0}e^{rt}}{K-\mu_{0}+{\mu_{0}e}^{rt}}.$$

Taking the second derivative of expression (13), we will obtain

(18) $$\mu^{\prime \prime }=\mu^{\prime }\cdot \left(r-\frac{2\mu r}{K}\right).$$

It follows that the value of the inflection point of the function graph is $\mu_{infl}=\frac{K}{2}$. The coefficient *K* is determined from the function graph. After finding $\mu_{infl}$, we substitute this value into expression (16) to obtain the value of the time at which the viscosity of the hydrogel $\mu =\mu_{infl}.$

(19) $$t_{infl}=\frac{1}{r}ln\left(\frac{K-\mu_{0}}{\mu_{0}}\right).$$

Expression (19) can be used to estimate the value of the parameter *r*, since the parameters K and $t_{0}$ are determined from the graphs, and $\mu_{0}$ is obtained experimentally. Based on the obtained dependencies, it can be concluded that the function (17) has an inflection point at $\left(\frac{K}{2};\;\frac{1}{r}ln\left(\frac{K-\mu_{0}}{\mu_{0}}\right)\right)$. Knowing the value of time $t_{infl}$, we can find *r.*

**Figure 8:**
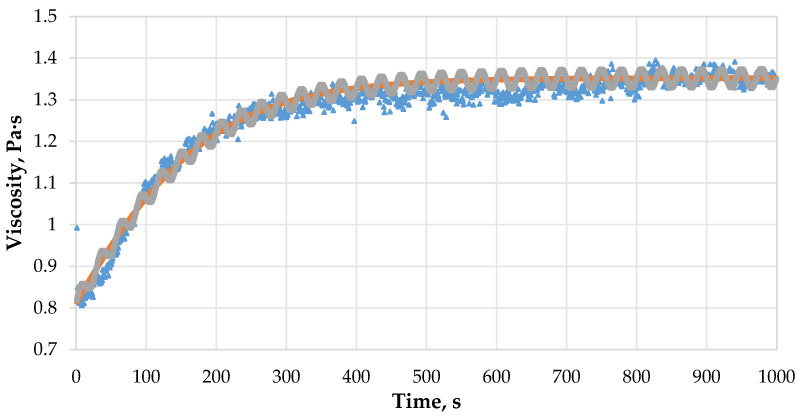
Comparison of gelation process models: experimental data (blue points), model according to the Verhulst equation (orange curve) and model according to the Verhulst equation with correction coefficient (grey curve).

In its explicit form, the Verhulst equation describes a smooth curve, each successive value of which is greater than the previous one. In our case, the viscosity values change according to an oscillatory law relative to a smoothly increasing curve. Therefore, it is necessary to introduce a correction expression that will describe the oscillatory behavior of the gel (Figure 8):

(20) a·sin(bt),

where a—amplitude of oscillations, Pa·s; b—a parameter, numerically equal to $\frac{2\pi }{T}$, where T is the oscillation period, 1/s.

Summarizing the above data, a mathematical model can be formulated that describes the dependence of the viscosity of the crosslinked polymer composition on time:

(21) $$\mu =\frac{\mu_{0}Ke^{rt}}{K-\mu_{0}+\mu_{0}e^{rt}}+a\cdot \sin\left(bt\right).$$

To quantitatively assess the degree of correspondence between the mathematical model and real experimental data, it is necessary to determine the measure of statistical relationship between the actual and calculated values (Table 1). The most informative indicator in this case is the correlation coefficient R, which can be calculated based on the following relationship:

(22) $$R=\sqrt{1-\frac{\sum {\left(y_{i}-{\hat{y}}_{i}\right)}^{2}}{\sum {\left(y_{i}-\frac{1}{n}\sum y_{i}\right)}^{2}}},$$

where $y_{i}$—actual viscosity value at the i-th second; ${\hat{y}}_{i}$—calculated viscosity value at the i-th second.

After analyzing the experimentally obtained data and calculating the missing coefficients, a table of values for shear rate (*γ*), oscillation amplitude (*a*), steady viscosity value (*K*), gelation time (*t_g_*), and parameters *b* and *r* was compiled (Table 2). For these parameters, power-law dependences on the shear rate were obtained, which were derived using approximation methods in Microsoft Excel and PlanetCalc.

The dependencies of the oscillation amplitude and parameter *b* on shear rate have been obtained. As the shear rate increases, the bond stresses in the molecules of the crosslinked polymer gel increases [38,39]. Consequently, the frequency of crosslinking-breakup reactions of polymer molecules and the limiting viscosity value will increase (due to the non-Newtonian behavior of the fluid) [30]. This leads to an increase in the amplitude of oscillations (parameter a) and a decrease in the oscillation period.

The power-law dependences of the parameters showing changes in viscosity and the rate of viscosity growth up to the moment of gelation are established. At high shear rates the resistance factor of polymer solution molecules increases, leading to a higher bond stress in the molecules, which hinders the further crosslinking process and the formation of more branched and larger molecules [40]. At low shear rates, the opposite trend is observed—the formation of polymer molecules with a high molecular weight—and as a result, an increase in the viscosity of the system. It follows that as the shear rate increases, the set viscosity value decreases (coefficient *K*) and the rate of increase in viscosity increases (coefficient *r*), and as the shear rate decreases, *K* increases and *r* decreases.

During the study of crosslinked polymer systems, the dependence of gelation time on shear rate was established experimentally.

Since at high shear rates the gel consists of polymer molecules with a low degree of polymerization [41], the formation time of a stable system under these conditions (gelation time) will be shorter. The opposite dependence is observed at low shear rates: the crosslinked polymer system will consist of more massive polymer molecules, which means that the polymerization (crosslinking) reaction of these compounds will proceed more slowly, which will lead to an increase in gelation time.

### 2.3. Study of Self-Oscillations in the Polymer Crosslinking Process

The issue of self-oscillations occurring during crosslinking of the polymer system requires special attention. Such behavior of polymer compositions can be caused by both characteristics of the studied solutions and instrumental reasons. Thus, the scientists Skvortsov I.Y., Malkin A.Y. and Kulichikhin V.G. [42] identify three causes of self-oscillations in gels:

- The first cause of oscillations in the low shear stress region in rotational rheometers is related to software features. Oscillations during shear deformations of Newtonian fluids occur at stress levels around 0.1 Pa. In a viscoelastic medium, this stress threshold can be significantly higher. In the case of crosslinked polymer system research, the stress variation range was from 4 to 7 Pa;
- The second type of oscillations is caused by surface friction at the contact between the solid boundary surface and the viscoelastic medium, leading to a slip–adhesion effect. This is a result of interaction between the studied sample and the measuring instrument, reflecting a real physical phenomenon of periodic slip–adhesion. However, the resulting oscillations are not related to the bulk rheological properties of the polymer system. This gel behavior can reflect and explain its actual behavior during movement through the manifolds, tubing and casing;
- The third type of self-oscillations appears as a result of volumetric bifurcations and transitions between several possible structural states of the system under shear deformations. In the studied composition, as described in the review, structure formation is caused by intramolecular and intermolecular crosslinking, with intermolecular crosslinking prevailing in terms of viscosity buildup intensity. Such gel self-oscillations can be explained by this behavior.

**Figure 9:**
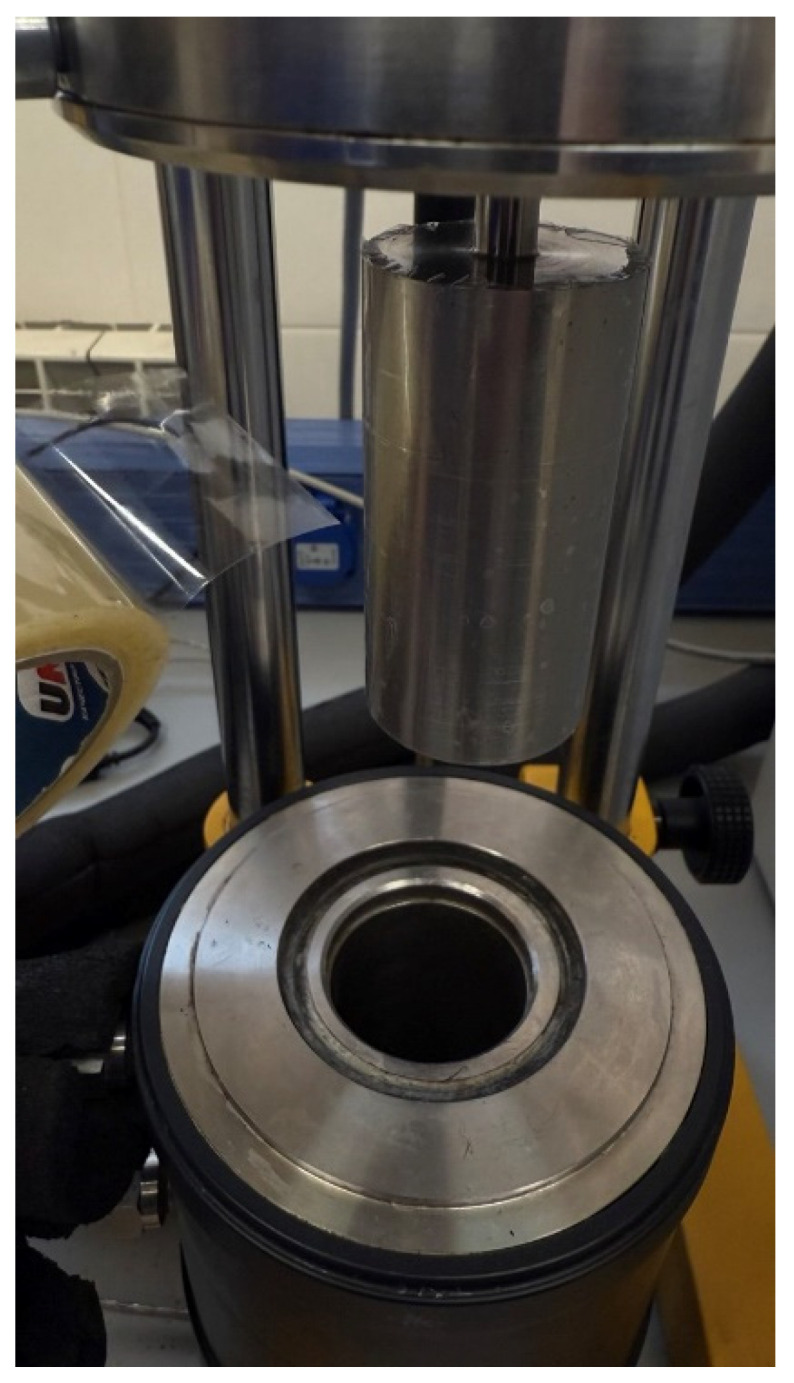
Measuring cylinder with taped surface insulation.

While the first and third causes of self-oscillations were explained through theoretical and rheological studies, the second cause of viscosity (shear stress) fluctuations requires additional experimental data validation. For this purpose, rheological studies were conducted by measuring the viscosity curve over time with the measuring cylinder surface isolated with a single layer of tape. This was done because the polymer composition has good adhesion to metal, thus creating an adhesion effect (Figure 9). As a result of studying the crosslinked polymer system viscosity curves with and without isolation and processing the obtained data, it was found that the viscosity amplitude decreased: a 20% reduction in standard deviation and a 17% decrease in coefficient of variation (Table 3).

**Figure 10:**
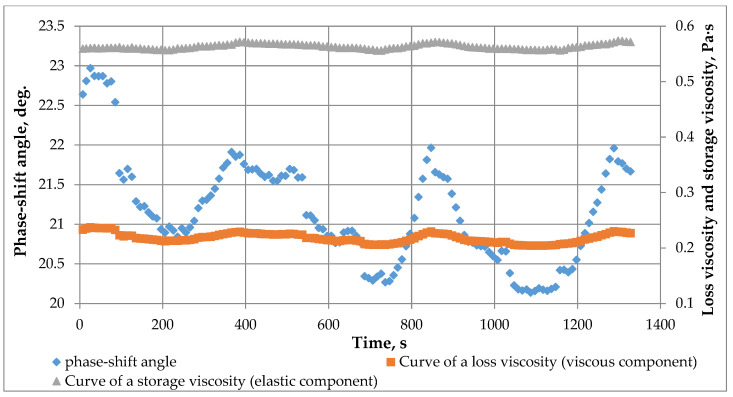
Results of rheological oscillation measurements on crosslinked polymer material.

On the other hand, when measuring gel formation kinetics at rest (under dynamic loads), the absence of viscosity oscillations was observed. Therefore, it was proposed that we conduct an oscillatory gel test at a frequency of f = 1 Hz and stress amplitude G = 10 Pa (Figure 10). The overall dynamics of the phase angle (the smaller the phase angle, the greater the elastic component of viscosity) is directed towards reduction, which correlates with the crosslinking process and the formation of a more elastic rather than viscous fluid. However, Figure 10 shows that the phase angle itself also exhibits oscillatory changes.

Thus, the self-oscillations of the effective viscosity of the crosslinked polymer system are partly related to the second reason, i.e., the adhesion process. However, it was not possible to completely eliminate this effect. Consequently, the existing reasons affecting the occurrence of viscosity self-oscillations also include the peculiarities of gel structure formation, which is a mixture of PHPAN and chromium (III) acetate.

**Figure 11:**
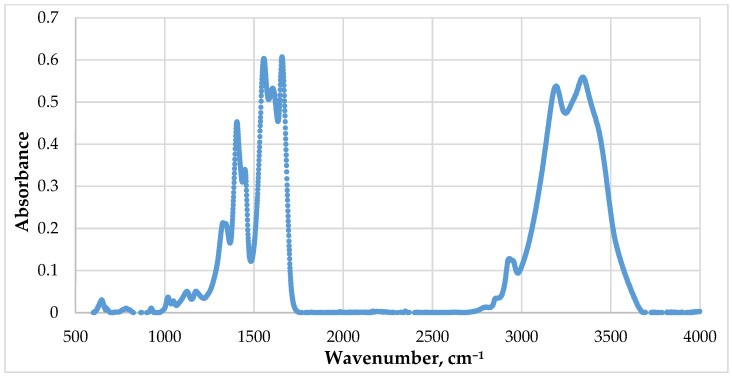
Infrared spectrum of gel formed from PHPAN and chromium (III) acetate.

In order to study the chemical bonds formed during crosslinking, an IR spectrum of the gel was recorded (Figure 11), which reveals several significant regions:

- Hydroxyl group region (3600–3200 cm^−1^);
- C-H region (3000–2800 cm^−1^);
- Carbonyl and carboxylate region (1700–1500 cm^−1^);
- Low-frequency vibrations region (1200–1000 cm^−1^).

The range of 1550–1650 cm^−1^ is of particular interest from a crosslinking process perspective, as it shows a shift in the amide (I) band and a broadening of the amide (II) band. These changes in the absorption spectra may indicate the formation of coordination bonds where Cr^3+^ ions act as electron pair acceptors, and nitrogen and oxygen atoms from amide groups (-CONH_2_) and PHPAN act as donors. When incomplete substitution of the acetate group occurs, which is observed in the spectrogram at 1400 cm^−1^ and higher wavenumbers, ionic bonds may form between acetate groups and chromium Cr^3+^, as well as with protonated PHPAN groups (-NH_3_^+^). The appearance of absorption bands in the spectrum below 600 cm^−1^ may indicate the formation of covalent Cr^3+^ bonds through oxygen.

Consequently, self-oscillations due to the third reason may be caused by reversible coordination and possibly ionic bonds. Furthermore, the sharp decrease in crosslinked polymer system viscosity in a narrow gap during rotation of the measuring system may be associated with mechanical destruction and irreversible rupture of covalent bonds.

**Figure 12:**
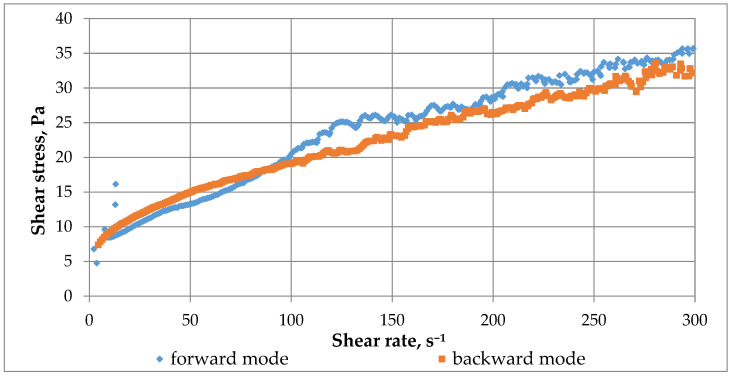
Hysteresis loop of crosslinked polymer system.

Finally, to determine the possibility of restoring the destroyed gel structure due to the presence of coordination or ionic bonds, an assessment of the gel’s thixotropy was conducted by recording a hysteresis loop (Figure 12). The gel formed after crosslinking was placed in a rotational viscometer cup and subjected to a test consisting of three modes: forward run with shear rate from 0 to 300 s^−1^, holding at shear rate 300 s^−1^, reverse run with shear rate from 300 to 0 s^−1^. Based on the hysteresis loop results, the presence of thixotropic behavior in the gel was established: during the reverse run, the shear stress decreases but not along the same trajectory as in the forward run, forming a loop up to a shear rate of approximately 100 s^−1^. Subsequently, in the range from 100 to 0 s^−1^, structure recovery occurs and there is almost complete coincidence of the shear stress curves (with the reverse run curve being slightly higher). Thus, the studied crosslinked polymer system possesses reversible bonds, most likely coordination bonds.

### 2.4. A Model of the Gelation Process in Narrow-Gap-Simulating Porous Media

**Figure 13:**
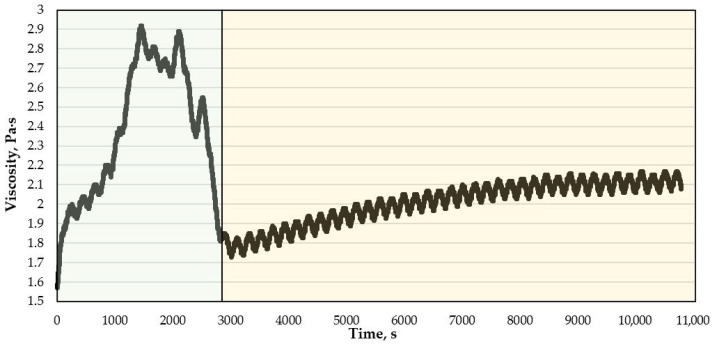
Dependence of the viscosity of a gel with a PHPAN concentration of 0.7% and a CA concentration of 0.07% flowing with a shear rate of 3.82 $s^{-1}$ in a 0.1 mm gap on time.

The behavior of the gel in the porous media differs from the behavior of the gel in the free volume (Figure 13).

The PC-GEL model and the model based on the Verhulst equation with the correction term (21) are unable to describe the gelation process in the porous media [13]; therefore, an alternative model is needed to describe the flow of the gel in the porous media.

In the process of analyzing the data on the gelation time and the viscosity parameter from time in the porous media, a model was obtained describing the process of crosslinking, bond breaking and reaching a steady viscosity value. The gelation process was divided into two parts.

In the first part, the viscosity value rises and falls to the initial viscosity value. This process can be approximated by the equation

(23) $$\frac{c\left(t+\frac{c}{\mu_{0}}\right)}{t!},$$

where c—the parameter responsible for the rate of viscosity growth; $\mu_{0}$—the value of viscosity at the moment *t* = 0.

In the second part, a smooth increase in viscosity values with pronounced oscillations is observed until a steady-state viscosity is reached. This part can be approximated by the Verhulst equation with a correction term. However, it is necessary to account for the shift in the equation of the second part so that it starts to influence the system toward the end of the first part. The equation used for the first part will not influence the second part, as its values approach to 0 in the second part.

(24) $$\frac{K\mu_{0}^{*}e^{r\left(t-t_{0}\right)}}{K-\mu_{0}^{*}+\mu_{0}^{*}e^{r\left(t-t_{0}\right)}}+a\sin\left(b\cdot t\right),$$

where $\mu_{0}^{*}$—the viscosity value at the moment of transition from the first to the second part of the gelation process (the moment of transition from bond breaking to smooth bond crosslinking), Pa·s; $t_{0}$—the moment in time when the system transitions from bond breaking to a smooth increase in viscosity, s.

Thus, a dependency was obtained that describes the behavior of the gel in the porous media

(25) $$\mu =\frac{c\left(t+\frac{c}{\mu_{0}}\right)}{t!}+\frac{K\mu_{0}^{*}e^{r\left(t-t_{0}\right)}}{K-\mu_{0}^{*}+\mu_{0}^{*}e^{r\left(t-t_{0}\right)}}+a\sin\left(b\cdot t\right).$$

The gel behavior model, described by Equation (25), demonstrates the physical processes of gelation both in the porous media and in the free volume (with c = 0, $t_{0}$ = 0).

The reaction rates of crosslinking and destruction, as well as the maximum viscosity value during the first stage, depend on the concentrations of PHPAN and CA. Additionally, the diameter of the pore involved in filtration plays an important role.

For a gel with a 4% PHPAN concentration and a 0.4% CA concentration, flowing with a shear rate of 3.82 $s^{-1}$ through a pore with a diameter of d = 0.5 mm, the second stage begins at t = 750 s.

**Figure 14:**
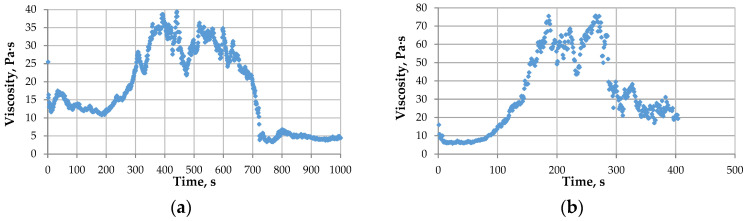
Dependence of the viscosity of a gel with a PHPAN concentration of 4% and a CA concentration of 0.4% flowing with a shear rate of 3.82 $s^{-1}$ through a pore d = 0.5 mm (**a**) and d = 1 mm (**b**) on time.

For the gel (with all other parameters being equal) flowing through a pore with a diameter of d = 1 mm, the first stage is shorter in duration and characterized by the highest viscosity value. This is due to the increased volume in which the gelation process occurs (Figure 14).

**Figure 15:**
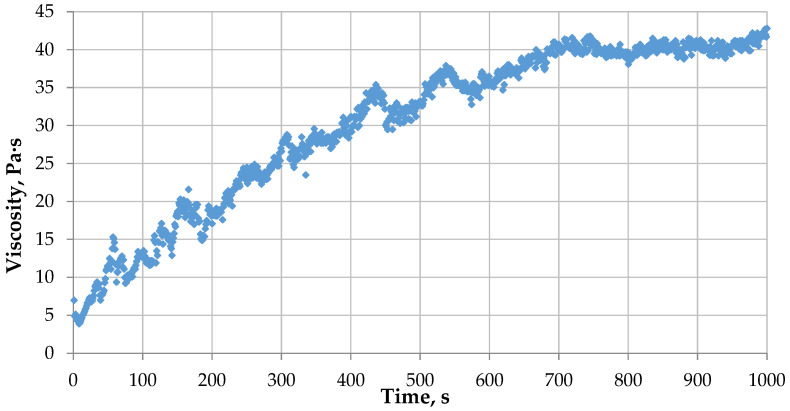
Dependence of the viscosity of a gel with a PHPAN concentration of 4% and a CA concentration of 0.4% flowing with a shear rate of 3.82 $s^{-1}$ through a pore d = 2 mm on time.

When the critical pore diameter is reached, the gelation process includes only the second stage. For example, for a gel with a 4% PHPAN concentration and a 0.4% CA concentration, flowing with a shear rate of 3.82 $s^{-1}$, the behavior, as in free volume, begins with a pore diameter of d = 2 mm. In this case, the first stage is not observed, and the graph can be described by the Verhulst limited growth model, applicable to free volume (Figure 15).

## 3. Conclusions

Currently, there is a wide range of software products designed for the digital modeling of physicochemical processes occurring in reservoirs during the application of crosslinked polymer gels for controlling filtration flows in heterogeneous reservoirs. The main difference between these programs lies in the variety of analytical models used to describe the physicochemical properties of gels. However, many aspects of modeling the physicochemical properties of the described compositions, such as the influence of component concentration on reaction kinetics, pore diameter effects, the influence of adsorption processes on gel formation kinetics, and analytical calculation of the porous media inaccessible to polymer molecule filtration, still require further research.

Comprehensive studies have established the causes of effective viscosity self-oscillations both in free volume and in porous media. The mechanism of gel adhesion to the metal part of the measuring cylinder has been clarified, and self-oscillations have been partially suppressed by taping the measuring cylinder. Self-oscillations arising due to bifurcations and the formation of new structures may be caused by reversible coordination and possibly ionic bonds. The subsequent sharp decrease in crosslinked polymer system viscosity in a narrow gap during rotation of the measuring system may be associated with mechanical destruction and the irreversible rupture of covalent bonds. The formation of these bonds was confirmed by IR spectroscopy of the obtained gels.

A more accurate gel formation kinetics model (Scott model) has been proposed, which improved the correlation coefficient for the studied samples from 0.76…0.81 to 0.95…0.97.

Experimental studies have revealed a correlation between shear rate and two key parameters: oscillation amplitude (*a*) and coefficient *b*. A direct dependence of molecular bond stress in the crosslinked polymer gel is observed with increasing shear rate, consequently intensifying the processes of intermolecular bond formation and rupture. This leads to an increase in the limiting viscosity value (as a manifestation of non-Newtonian fluid properties), resulting in increased oscillation amplitude and decreased oscillation period.

Power-law dependencies have been established for parameters describing viscosity changes and viscosity growth rate before gel formation. At high shear rates, increased resistance of crosslinked polymer system molecules is noted, along with higher intermolecular bond stress and hindered crosslinking processes, limiting the formation of branched macromolecules. Conversely, at low rates, the opposite effect is observed, associated with the formation of high-molecular structures and significant gel viscosity growth.

The proposed mathematical model demonstrates universality in describing the viscosity behavior of the crosslinked polymer composition, effectively characterizing gel behavior both in confined narrow-gap-simulating porous media and in free volume (at zero values of parameters c and $t_{0}$). The crosslinking rate, maximum viscosity value, and degradation rate at the first stage of gel formation depend on the PHPAN and chromium (III) acetate concentrations.

Experimental studies have established that the first stage of gel formation, characterizing gel behavior in confined narrow-gap-simulating porous media, manifests itself within a certain range of pore diameter values. For a crosslinked polymer composition with 4% PHPAN and 0.4% chromium (III) acetate concentrations at a shear rate of 3.82 s^−1^, free-volume behavior without the first stage of gel formation is observed starting from a pore diameter of 2 mm.

## 4. Materials and Methods

The gelation process can be described by studying the rheological characteristics of the polymer composition in accordance with RD-39-8148311-206-85, where, in recent times, ultra-precise rotational viscometers have been used instead of screen viscometers [43,44]. For the purposes of rheological analysis, a polymer composition widely utilized in flow-deviation technologies was prepared, comprising an aqueous solution of PHPAN and CA, with concentrations of 0.7% and 0.07%, respectively [45,46,47].

Over a period of 3.5 h, viscosity measurements of the initial solution were conducted at one-second intervals using the RHEOTEST rotational viscometer (Rheotest Messgerate Medingen GmbH, Ottendorf-Okrilla, Germany). The measurements were taken at the following shear rates, determined in accordance with the simplified Formulas (26) and (28): 384 s^−1^, 76 s^−1^, 15.28 s^−1^, 7.6 s^−1^, and 3.82 s^−1^. These values correspond to the shear rates of the polymer system within the tubing string and in the porous media at distances of 5 m, 26 m, 52 m, and 103 m from the wellbore, respectively. The data used for the shear rate calculations are provided for an “average” well in Table 4 and Table 5.

The shear rate was calculated using a simplified equation [48]:

(26) $$\gamma_{TS}=\frac{8\vartheta_{TS}}{d_{TS\;int}},$$

where $\gamma_{TS}$—shear rate in the tubing string, 1/s; $\vartheta_{TS}$—flow velocity in the tubing string, m/s; $d_{TS\;int}$—internal diameter of the tubing string, m.

The flow velocity in the tubing string is

(27) $$\vartheta_{TS}=\frac{4Q}{\pi d_{TS\;int}^{2}},$$

where Q—volumetric flow rate of polymer solution injection, m^3^/s.

The following simplified formulas were used to calculate the filtration velocity and shear rate in the porous media at a distance *r* from the tubing string [49]:

(28) $$\gamma_{pore}=\frac{8\vartheta_{filtration}}{d_{av\;pore}\cdot m},$$

(29) $$\vartheta_{filtration}=\frac{Q}{2\pi rh},$$

where $\gamma_{pore}$—shear rate in the porous media, 1/s; $\vartheta_{pore}=\frac{\vartheta_{filtration}}{m}$—actual seepage velocity in the porous media, m/s; $d_{av\;pore}$—average pore diameter of the reservoir, m; h—thickness of the examined reservoir formation, m; m—connected porosity.

At the same time, more accurate models can be used to calculate the shear rate in the reservoir rock, such as the Hirasaki–Pope Equation (30) and the Chauveteau–Zaitoun Equation (31), which take into account the reservoir properties (porosity and permeability) [17,50]:

(30) $$\gamma =F\left(\frac{\vartheta_{pore}}{\sqrt{km}}\right),$$

(31) $$\gamma =F\left(\frac{\vartheta_{pore}\left(1-m\right)}{\sqrt{km}}\right),$$

where F—flow resistance factor; k—absolute permeability of the reservoir rock, µm^2^; m—connected porosity.

A calculation of the permeability values was conducted using expression (32), and the average porosity value was derived based on the empirical relationship with the average permeability, as per Equation (33):

(32) $$k=\frac{{d^{2}}_{av\;pore}}{32}=0.028125\;µm^{2},$$

(33) $$m=\frac{\log28.125+2.77}{0.218}:100=0.1935$$

Using Darcy’s Equation (34), the flow resistance factor will be calculated according to Equation (35):

(34) $$dP=\frac{\vartheta \cdot L\cdot \mu }{k},$$

(35) $$F=\frac{dP_{solution}}{dP_{water}}\approx \frac{\mu_{solution}}{\mu_{water}},$$

where $dP_{solution}$—the pressure drop during the filtration of a polymer solution, Pa; $dP_{water}$—the pressure drop during water filtration, Pa; $\mu_{solution}$—viscosity of a polymer solution, Pa·s; $\mu_{water}$—viscosity of water, Pa·s.

The following viscosity values for water and the polymer solution were used in the calculation (Pa·s):

$$\mu_{water}=10^{-3};\;\mu_{solution\;1}=\mu_{0};\;\mu_{solution\;2}=K;\;\mu_{solution\;3}=\frac{K}{2},$$

where $\mu_{0}$—viscosity of the hydrogel at the beginning of the experiment, Pa·s; K—stable viscosity value, Pa·s (Table 6).

The shear rate values calculated using Equations 28, 30, and 31 (Table 6) exhibit differences of approximately 3 to 20 times. In this study, shear rates determined using the simplified equation were employed. These calculations are sufficient for examining the dynamics of the gelation process in crosslinked polymer systems within free volume, as adherence to a specific shear rate is not a requisite. All measurements were performed under standard conditions.

The gelation process of the crosslinked polymer system in the porous media can currently be described using the PC-Gel software based on Kenneth Sorbie’s method [13,51], utilizing the dependence of the crosslinked polymer system’s viscosity on concentration and the gelation constant.

PC-Gel may be classified among specialized computational tools designed to simulate physicochemical processes associated with polymer gel systems, particularly those used in flow diversion and conformance control applications. Such simulators typically implement analytical or semi-empirical constitutive models describing gelation kinetics, viscosity evolution, and the development of gel network structures under specified thermochemical conditions. Rather than representing a general-purpose reservoir simulator, PC-Gel is used to analyze the behavior of gel-forming compositions through parameterized kinetic and rheological relationships.

The functional principles underlying PC-Gel are consistent with those employed in other modeling environments developed for polymer gel systems, where gelation is described via effective kinetic constants and temperature-dependent rate expressions. In particular, Arrhenius-type formulations are widely adopted for representing the temperature sensitivity of gelation rates.

In the Scott model, the Arrhenius equation [13] is used to calculate the gelation constant, which has the following form:

(36) $$K_{s}=A\cdot \exp\left(\frac{E_{a}}{RT}\right)$$

However, this model does not allow determination of the gelation reaction constant, as it contains two unknown parameters: the pre-exponential factor (Arrhenius constant) *A* and the activation energy $E_{a}$. Additionally, it does not provide a methodology for their determination.

In the present study, all experiments were conducted under isothermal conditions, and no systematic temperature variation was performed. Consequently, the dataset does not permit reliable determination of the activation energy via Arrhenius-type analysis. This represents one of the limitations of the methodology employed in this work.

Importantly, the Arrhenius equation was introduced in this work not as a basis for thermodynamic parameter estimation, but as part of the mathematical formalism underlying the Scott model used for comparative analysis. The Scott approach employs an Arrhenius-type representation of the gelation rate constant to describe the temperature dependence of effective kinetic parameters. In this context, the use of the Arrhenius equation in our work serves a methodological purpose, enabling comparison between gelation constants obtained via the proposed experimental–analytical procedure and those defined within the Scott modeling framework.

From a physicochemical standpoint, the theoretical applicability of Arrhenius-type behavior to chromium(III)-mediated gelation systems remains well justified. Gelation in such systems is controlled by coordination interactions and ligand substitution reactions involving Cr^3+^ complexes, whose kinetics are inherently thermally activated. This interpretation is consistent with established literature on chromium-crosslinked polymer gels [31,32], where Arrhenius-type dependencies describe effective macroscopic parameters rather than elementary reaction barriers.

In this regard, it is proposed to determine the gelation constant using the effective concentrations method. The experimental procedure consists of the following stages:

- Determination of the optimal wavelength for assessing changes in the optical density of crosslinked polymer system;
- Evaluation of changes in the optical density of the crosslinked polymer system over time Dg = f(t);
- Calculation of gel concentration according to the Beer–Bouguer–Lambert (BBL) extinction law, description of the curve C_g_ = f(t) by an equation and linearization of the curve, and estimation of the gelation constant K_g_;
- Construction of the viscosity–time curve using the Scott model.

To compare the obtained results on the time-dependent viscosity changes in the crosslinked polymer system in the porous media, a series of experiments was conducted to record the gelation curve of the crosslinked polymer system in a narrow gap of varying sizes [52,53]. For this purpose, the crosslinked polymer composition was placed in an Anton Paar MCR 102 modular rheometer (Anton Paar GmbH, Austria), and the gel viscosity values were measured at 1 s intervals over a period of 3.5 h.

The methodology for conducting rheo-viscometry studies of crosslinked polymer systems in narrow gaps consisted of the following steps:

1. Preparation of crosslinked polymer system. Two solutions were prepared separately: an aqueous polymer solution and an aqueous solution of chromium (III) acetate. The required amount of polymer, weighed on an analytical balance according to the specified concentration, was dissolved in distilled water using an overhead stirrer for 1 h at 350 rpm at room temperature. Thereafter, after preparation, the uniformly mixed polymer solution was placed into the rheometer cup, the aqueous chromium(III) acetate solution was added, and rheological experiments were conducted at a temperature of 20 °C.
2. Rheological studies of the crosslinked polymer system at a specified constant shear rate CR = const and at a given gap width (ranging from 0.1 mm to 2 mm);
3. Data processing using the Verhulst logistic equation.

This approach allowed for comprehensive analysis of the rheological behavior of the crosslinked polymer system under controlled experimental conditions.

## Figures and Tables

**Table 1:** Comparison of process gelation model correlations based on the correlation coefficient at different shear rates.

№	Shear Rate, 1/s	$R_{V.}$ *, %	$R_{corr.\;exp.}$ **, %	$R_{PC-GEL}$ ***, %
1.	3.82	90.1	96.2	79.0
2.	7.60	89.6	95.6	80.1
3.	15.28	86.6	94.8	75.6
4.	76.00	91.2	96.1	77.8
5.	384.00	88.9	97.3	81.2

* $R_{V.}$—correlation based on the Verhulst Equation (17). ** $R_{corr.\;exp.}$—correlation based on the Verhulst equation with correction expression (21). *** $R_{PC-GEL}$—correlation based on Equation (12).

**Table 2:** Values and dependencies of the correlation parameters of the Verhulst equation with correction expression (21) on shear rate.

№	*γ*, 1/s	*a*, 1/s		*b*, 1/s		*K*, Pa·s		*r*, 1/s		*t_g_*, s	
1.	3.82	0.0222	a=0.03γ ^−0.63^	0.22	b=0.17γ ^0.23^	1.352	K=0.17γ ^−0.62^	0.009	r=0.17γ ^0.47^	985.5	*t_g_* = 2166*γ*^−0.52^
2.	7.60	0.0092		0.32		0.622		0.012		915.5	
3.	15.28	0.0055		0.35		0.458		0.022		397.5	
4.	76.00	0.0014		0.40		0.191		0.035		303.5	
5.	384.00	0.0012		0.70		0.066		0.082		83.5	

**Table 3:** Comparative statistical processing of crosslinked polymer system rheological measurements: insulated and non-insulated cylinder.

№	Parameter	Tape-Insulated Cylinder	Cylinder
1.	Standard deviation, Pa·s	0.0124	0.0154
2.	Coefficient of variation, %	0.633	0.7654

**Table 4:** Experimental conditions for the injection of the crosslinked polymer system into the tubing string.

№	Parameters	Units of Measurement	Value	
			Parameters of Cement Pumping Unit	
			2nd Transmission d_bushing_ = 100 mm	4th Transmission, d_bushing_ = 100 mm
1.	Injection flow rate of the crosslinked polymer system	L/s	3	9
2.	Experiment time	min	33.5	11.2
3.	Shear rate	1/s	127.7	383.1
4.	Temperature	°C	20–34.7	20–29.6
5.	Temperature gradient	°C/min	0.44	0.86

**Table 5:** Well parameters and geological–physical conditions.

Well Parameters and Geological–Physical Conditions of the Productive Formation		Units of Measurement	Value
Wellhead temperature		°C	20
Reservoir temperature		°C	40
Temperature gradient		°C/min	0.11
Nominal diameter of the tubing string (*d_TSnom_*)		mm	73
Internal diameter of the tubing string (*d_TSint_*)		mm	62
Nominal diameter of the production casing (D_PCnom_)		mm	178
Internal diameter of the production casing (D_PSint_)		mm	160
Well depth (L_well_)		m	2150
Depth of tubing string descent (h_TS_)		m	2000
Injection flow rate (Q) of the crosslinked polymer system with the cementing unit	2nd transmission, d_bushing_ = 100 mm	L/s	3
	4th transmission, d_bushing_ = 100 mm	L/s	9
Formation thickness, h		m	5
Radius of the feeding contour (radial extent), R_fc_		m	250
Average pore diameter		µm	30

**Table 6:** Shear rate values in the porous media.

Distance from the Tubing String, m	Viscosity of Polymer Solution, Pa·s	Simplified Equation (*γ*), s^−1^	Hirasaki–Pope Equation (*γ*), s^−1^	Chauveteau–Zaitoun Equation (*γ*), s^−1^
5	*µ* = *K* = 0.1911	76.00	3.82	3.08
	*µ* = *K*/2 = 0.09555		1.91	1.54
	$\mu =\mu_{0}$ = 0.1475		2.94	2.38
26	*µ* = *K* = 0.4523	15.28	1.82	1.46
	*µ* = *K*/2 = 0.22615		0.91	0.73
	$\mu =\mu_{0}$ = 0.3112		1.25	1.01
52	*µ* = *K* = 1.2829	7.60	2.56	2.07
	*µ* = *K*/2 = 0.64145		1.28	1.03
	$\mu =\mu_{0}$ = 1.1486		2.29	1.85
103	*µ* = *K* = 1.3241	3.82	1.33	1.07
	*µ* = *K*/2 = 0.66205		0.66	0.54
	$\mu =\mu_{0}$ = 0.9928		1.00	0.80

## Data Availability

Data is contained within the article.

## Abbreviations

The following abbreviations are used in this manuscript:

PHPAN	Partially hydrolyzed polyacrylonitrile
CA	Chromium acetate
BBL	Beer–Bouguer–Lambert
